# Whole-genome sequencing-based tumor-informed circulating tumor DNA monitoring for early detection of recurrence in patients with solid tumors

**DOI:** 10.1093/oncolo/oyag404

**Published:** 2026-10-16

**Authors:** Mridula George, Gary S Schwartz, Wassim McHayleh, Vitaly Margulis, Hideaki Bando, Takayuki Yoshino, George Ansstas, Carly Bess Scalise, Sumedha Sudhaman, Arkarachai Fungtammasan, Bharat Sridhar, Jordan Feeney, Himanshu Sethi, Morgan M Quail, Sharlene Velichko, Wilson Tan, Alexander Hsieh, Anastasia Butskova, Ryan Ptashkin, Carcia Carson, Ashley Wray, Charuta C Palsuledesai, Meenakshi Malhotra, Ahmet Zehir, Eser Kirkizlar, Matthew Rabinowitz, Ryan K Swenerton, Gabrielle Heilek, Adham Jurdi, Adam C ElNaggar, Michael Krainock, Caitlin A Schonewolf, Angel A Rodriguez, Shruti Sharma, Ekaterina Kalashnikova, Minetta C Liu, Alexey Aleshin, Matthew D Galsky

**Affiliations:** Breast Cancer Center, Rutgers Cancer Institute, New Brunswick, NJ 08901, United States; Department of Thoracic Surgery & Lung Transplantation, Baylor University Medical Center, Dallas, TX 75246, United States; Hematology and Medical Oncology, Advent Health Cancer Institute, Altamonte Springs, Orlando, FL 32804, United States; Urologic Oncology, UT Southwestern Medical Center, Dallas, TX 75235, United States; Department of Gastrointestinal Oncology, National Cancer Center Hospital East, Kashiwa, Chiba 277-8577, Japan; Department of Gastrointestinal Oncology, National Cancer Center Hospital East, Kashiwa, Chiba 277-8577, Japan; Division of Oncology, Washington University School of Medicine in St. Louis, St Louise, MO 63110, United States; Oncology, Natera, Inc., Austin, TX 78753, United States; Oncology, Natera, Inc., Austin, TX 78753, United States; Oncology, Natera, Inc., Austin, TX 78753, United States; Oncology, Natera, Inc., Austin, TX 78753, United States; Oncology, Natera, Inc., Austin, TX 78753, United States; Oncology, Natera, Inc., Austin, TX 78753, United States; Oncology, Natera, Inc., Austin, TX 78753, United States; Oncology, Natera, Inc., Austin, TX 78753, United States; Oncology, Natera, Inc., Austin, TX 78753, United States; Oncology, Natera, Inc., Austin, TX 78753, United States; Oncology, Natera, Inc., Austin, TX 78753, United States; Oncology, Natera, Inc., Austin, TX 78753, United States; Oncology, Natera, Inc., Austin, TX 78753, United States; Oncology, Natera, Inc., Austin, TX 78753, United States; Oncology, Natera, Inc., Austin, TX 78753, United States; Oncology, Natera, Inc., Austin, TX 78753, United States; Oncology, Natera, Inc., Austin, TX 78753, United States; Oncology, Natera, Inc., Austin, TX 78753, United States; Oncology, Natera, Inc., Austin, TX 78753, United States; Oncology, Natera, Inc., Austin, TX 78753, United States; Oncology, Natera, Inc., Austin, TX 78753, United States; Oncology, Natera, Inc., Austin, TX 78753, United States; Oncology, Natera, Inc., Austin, TX 78753, United States; Oncology, Natera, Inc., Austin, TX 78753, United States; Oncology, Natera, Inc., Austin, TX 78753, United States; Oncology, Natera, Inc., Austin, TX 78753, United States; Oncology, Natera, Inc., Austin, TX 78753, United States; Oncology, Natera, Inc., Austin, TX 78753, United States; Oncology, Natera, Inc., Austin, TX 78753, United States; Oncology, Natera, Inc., Austin, TX 78753, United States; Icahn School of Medicine at Mount Sinai, Mount Sinai Health System, New York, NY 10029, United States

**Keywords:** circulating tumor DNA, whole-genome sequencing, molecular residual disease, prognostic biomarkers, breast cancer, non-small-cell lung cancer, melanoma, colorectal cancer, renal cell carcinoma

## Abstract

**Background:**

Circulating tumor DNA (ctDNA) assays can transform patient management by enabling molecular residual disease (MRD) detection, prognosis prediction, and treatment monitoring. Here, we report clinical performance of Signatera Genome, a whole-genome sequencing (WGS)-based ctDNA assay, in a diverse pan-cancer cohort at landmark and longitudinal settings post-definitive treatment.

**Methods:**

We performed a retrospective analysis of 386 patients with one of the following cancers: breast, colorectal, non-small-cell lung, melanoma, or renal cell carcinoma. Signatera Genome assays, designed with high-quality variants selected from the matched tumor and normal WGS data, were used to detect ctDNA in the associated patients’ longitudinal plasma samples collected post-operatively. The correlation between ctDNA-based MRD status and systemic, distant recurrence-free survival (systemic DRFS) was assessed using Cox regression analysis.

**Results:**

In the pan-cancer cohort, landmark analysis at 3 months post-surgery revealed that patients with MRD-positivity had significantly inferior systemic DRFS compared to ctDNA-negative patients (hazard ratio [HR]: 23.7, *P* < .0001). Additionally, ∼30% improvement in 24-month systemic DRFS was observed among adjuvant-treated MRD-positive patients, whereas MRD-negative patients derived no clear benefit from adjuvant therapy. In the longitudinal setting, MRD positivity anytime post-definitive treatment surveillance significantly correlated with shorter systemic DRFS compared to serial MRD negativity (HR: 103.4, *P* < .0001). Tumor-specific analyses yielded similar findings. Notably, 62% (72/116) of patients had ctDNA detected in the ultra-sensitive range of <100 ppm.

**Conclusions:**

Our findings underscore the robust clinical performance of the Signatera Genome assay for MRD detection across solid tumors. Its broad applicability and high specificity support its integration into clinical practice and prospective trials.

Implications for practice This study shows that Signatera Genome, a whole-genome sequencing-based, bespoke circulating tumor DNA (ctDNA) assay, accurately detects molecular residual disease and identifies patients at high recurrence risk across various solid tumors. Post-treatment ctDNA detection strongly correlates with relapse, while patients with ctDNA positivity at landmark show meaningful benefit from adjuvant therapy. These results suggest ctDNA monitoring can personalize care by improving risk stratification, guiding treatment, and enabling early recurrence detection. Future prospective validation may support its inclusion in clinical guidelines for more effective cancer management.

## Introduction

Despite advances in multimodality therapy, disease recurrence remains a significant cause of morbidity and mortality across solid tumor types.^1^^,^^2^ Early detection of recurrence is crucial for initiating timely interventions to improve patient outcomes. Traditional surveillance methods, such as radiographic imaging and serum-based biomarkers, often lack the sensitivity and specificity required to detect residual disease or identify early recurrence post-definitive treatment, particularly in asymptomatic individuals. Furthermore, such modalities may detect recurrence when the volume of cancer cells has surpassed the threshold for curative treatment. The advent of circulating tumor DNA (ctDNA) assays has transformed the ability to detect molecular residual disease (MRD), predict patient prognosis, and monitor treatment response. Recent studies have demonstrated the clinical utility of ctDNA-based MRD detection for anticipating recurrence, tailoring surveillance strategies, and potentially guiding risk-adapted treatment decisions.^3–11^

Among various approaches, tumor-informed ctDNA assays have demonstrated superior performance in detecting MRD compared to plasma-only assays. Previously, Signatera, a clinically validated, personalized, tumor-informed 16-plex PCR-NGS ctDNA assay, was reported to offer high sensitivity and specificity for detecting MRD across several solid tumor types.^7^^,^^12–17^ Signatera relies on a proprietary approach to select a highly curated set of tumor variants from whole-exome sequencing (WES) data of matched tumor tissue and normal blood samples, followed by deep sequencing of plasma libraries at >100,000x per variant. The Signatera Genome assay employs whole-genome sequencing (WGS) to identify highly curated somatic variants for each patient’s tumor, enabling multiplex PCR-based tracking of ctDNA across a wider genomic footprint; this results in an assay with an enhanced ability to detect MRD in specific clinical scenarios, such as low-shedding tumors and earlier disease states.

While the potential of WGS-based ctDNA assays for MRD detection and relapse prediction is increasingly recognized in select individual tumor types, the broad clinical validation of WGS-based tumor-informed assays across various cancers has been limited. Therefore, we conducted a comprehensive retrospective pan-cancer analysis of Signatera Genome performance to evaluate its sensitivity and clinical performance in both landmark and longitudinal settings following definitive treatment.

## Materials and methods

### Patients and study design

For this pan-cancer retrospective analysis, we generated Signatera Genome results from de-identified, clinically annotated residual samples from commercial ctDNA testing with WES-based Signatera (Signatera, WES-based 16-plex PCR-NGS assay; Natera, Inc.) performed during 2020–2025 in patients with breast cancer, non-small-cell lung cancer (NSCLC), renal cell carcinoma (RCC), melanoma, and colorectal cancer (CRC). The key inclusion criteria were (1) availability of plasma timepoints collected post-surgery, during adjuvant therapy and surveillance; and (2) available clinical information (eg, dates of treatment/intervention and survival outcomes). Adjuvant treatment decisions and cadence of ctDNA testing were determined by the treating physician as part of routine clinical care. Clinical and pathological characteristics, including age, sex, histologic subtype, pathologic stage, adjuvant treatment, and survival outcomes, were abstracted retrospectively from Natera’s commercial database under an IRB-approved protocol (Protocol #23-067-GT; Salus IRB #24401). As a secondary analysis of existing data, this study was exempt from the requirement for written informed consent and conducted in accordance with the Declaration of Helsinki.

### Personalized, tumor-informed ctDNA assays

Signatera Genome assays were designed, consisting of somatic variants selected from the WGS data of tumor tissue and matched normal genomic (g)DNA biospecimens. In brief, gDNA isolated from FFPE tumor tissue and gDNA isolated from matched whole blood were sequenced on the NovaSeq-X platform (Illumina, Inc.). WGS data were evaluated for quality metrics and then processed through a proprietary somatic variant calling method and bioinformatics pipeline, resulting in a prioritized list of variants for designing PCR amplicons. Multiplexed targeted PCR (mPCR) was performed on cell-free (cf)DNA residual libraries available from commercially tested patients, followed by amplicon-based sequencing to track and quantify ctDNA. These assays detect plasma ctDNA using a sample calling strategy that integrates target confidences and sample-level noise into a final confidence score, without a minimum high-confidence variant cutoff. The ctDNA concentration was measured in mean tumor molecules (MTM)/mL plasma.

### Statistical analysis

Systemic distant recurrence-free survival (systemic DRFS) was defined as the time from surgery until the date of extracranial distant recurrence diagnosis. A Cox proportional hazards regression model was used to evaluate the association between ctDNA status and systemic DRFS. To account for heterogeneity in baseline hazard across cancer indications, a Gamma-distributed frailty term at the indication level was included in the Cox model, estimated via penalized partial likelihood. This random effect multiplicatively scales the baseline hazard per indication, yielding a ctDNA effect estimate adjusted for unmeasured between-indication variability. Frailty values ≥1 across indication groups confirm meaningful heterogeneity in baseline risk that is not explained by ctDNA status alone. Ignoring this would violate the assumption of a common baseline hazard and could bias the ctDNA effect estimate. Including the frailty term produces more realistic, indication-adjusted hazard ratios (HRs). Analysis at the post-surgical timepoint was landmarked at 3 months to address the immortal time bias, classifying a patient as MRD-positive if ctDNA was detected at any timepoint within those 3 months, regardless of the adjuvant treatment status (Figure 1A and B). The longitudinal, post-definitive treatment analysis included all timepoints collected after the end of adjuvant treatment (inclusive of radiotherapy, if administered) or after 3 months from the start of maintenance treatment, if given (Figure 1A and B). Patients with local/locoregional-only recurrences or brain metastases were excluded from sensitivity and specificity analyses in the landmark and longitudinal, post-definitive treatment settings.

**Figure 1.**
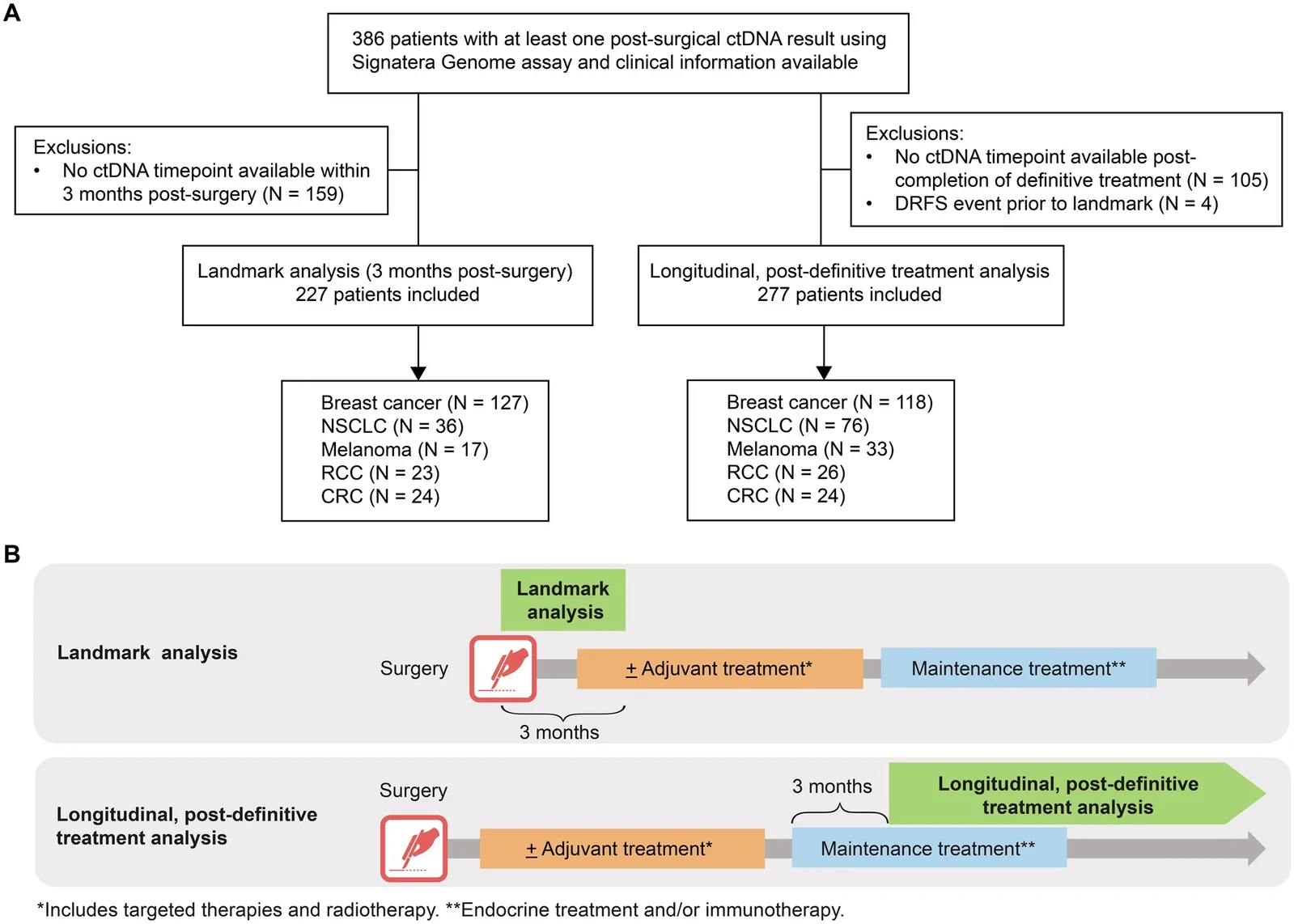
(A) CONSORT diagram depicting patient inclusion in the cohort and subanalyses. (B) Schema depicting time points included in the longitudinal, post-definitive treatment, and landmark analyses.

## Results

### Patient characteristics

A total of 2674 longitudinal plasma samples from 386 patients across 5 solid tumor types: breast cancer (227/386, 58.8%), NSCLC (76/386, 19.7%), melanoma (33/386, 8.5%), RCC (26/386, 6.7%), and CRC (24/386, 6.2%) were analyzed. Patient characteristics are summarized in Table 1. The median follow-up for the cohort was 22.5 months (range: 1.5-85.4), with a median of 6 (range: 1-35) ctDNA timepoints per patient. Of the total 2674 plasma samples analyzed, 390 samples corresponding to 116 unique patients were MRD-positive. ctDNA levels varied, with a substantial proportion of samples detected below 0.1 MTM/mL and/or 0.01% VAF (Figure 2). In particular, nearly half (169/390, 43%) of the samples (corresponding to 62% of unique patients [72/116]) were detected in the ultra-sensitive range of <0.01% VAF (ie, <100 ppm).

**Figure 2.**
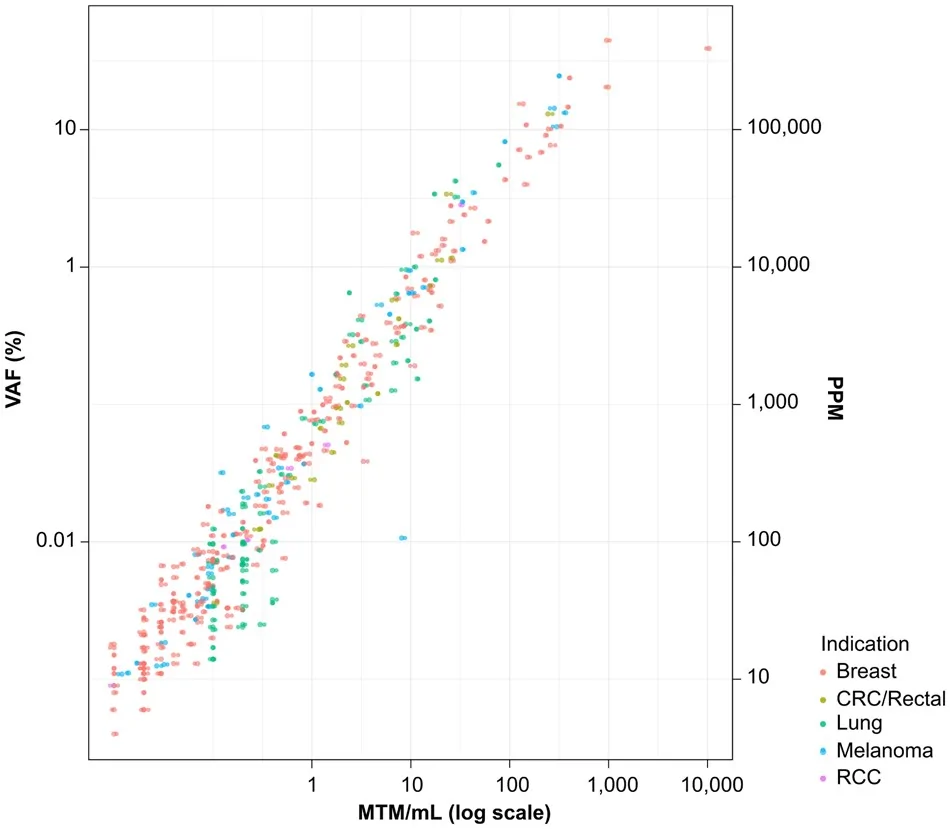
Correlation of ctDNA levels measured in mean VAF (%) with MTM/mL and PPM across MRD-positive samples (*n* = 390). Samples were bucketed and color-coded by tumor type. MTM, mean tumor molecules; PPM, parts per million; VAF, variant allele frequency.

**Table 1.** Patient and tumor characteristics (*N* = 386).

	All	Breast cancer	NSCLC	Melanoma	RCC	CRC
**No. of patients (%)**	386 (100%)	227 (58.8%)	76 (19.7%)	33 (8.5%)	26 (6.7%)	24 (6.2%)
**Age in years (range)**	55 (27-88)	55 (27-85)	69 (34-83)^a^	63 (32-83)	59 (20-78)	70 (44-88)
**Sex**						
** Male**	86	1	29	24	18	14
** Female**	298	226	45	9	8	10
** Not available**	2	–	2	–	–	–
**Stage**						
** I**	113	50	61	0	2	0
** II**	112	75	12	14	2	9
** III**	161	102	3	19	22	15
**Subtype **		TNBC (139, 61%)	Adenocarcinoma (58, 76.3%)		Clear cell (21, 80.8%)	
		HR+, HER2- (83, 37%)	Non-adenocarcinoma (18, 23.7%)		Non-clear cell (3, 11.5%)	
		HER2 + (5, 2%)			Other/unknown (2, 7.7%)	
**Recurrence**						
** No**	317	201	60	16	23	17
** Local**	20	9	6	4	1	0
** Systemic, distant **	49	17	10	13	2	7
**No. of ctDNA timepoints **	2,674	1,644	570	143	238	79
**No. of ctDNA timepoints per patient, median (range)**	6 (1-35)	7 (1-20)	7 (1-31)	4 (1-10)	4 (1-9)	3 (2-7)
**Median follow-up in months (range)**	22.5 (1.5-85.4)	20 (1.5-54.8)	40.8 (16.3-85.4)	27.2 (3.7-72)	28.4 (21-35.7)	30.7 (5.4-43)

a Age information not available for 5 patients.

### Prognostic and predictive value of MRD status at the landmark timepoint

We first evaluated whether the Signatera Genome assay can be used to detect MRD in the immediate post-operative window and inform patient prognosis. Landmark analysis performed at 3 months post-surgery (Figure 1B) demonstrated that patients with MRD positivity had significantly inferior systemic DRFS compared to MRD-negative patients (HR: 23.7; 95% CI, 8.7-64.5; *P* < .0001; Figure 3A), with a 36-month systemic DRFS of 35.3% (95% CI, 14.4-86.5%) vs 93.9% (95% CI, 90.0-97.9%), respectively. Furthermore, among patients with MRD positivity within 3 months of surgery, those receiving adjuvant therapy had a median systemic DRFS of 34.6 months compared to 8.8 months for those not receiving systemic treatment, although the difference was not significant due to low patient numbers (Figure 3B). Approximately 33% improvement in 12-month systemic DRFS was observed among adjuvant-treated MRD-positive patients (67.1% vs 33.3% for adjuvant-treated [*N* = 23] vs untreated [*N* = 10]). In contrast, among MRD-negative patients, no systemic DRFS benefit was observed in those receiving adjuvant therapy (24-month systemic DRFS: 91% vs 94.4% for adjuvant-treated [*N* = 104] vs untreated [*N* = 77]).

**Figure 3.**
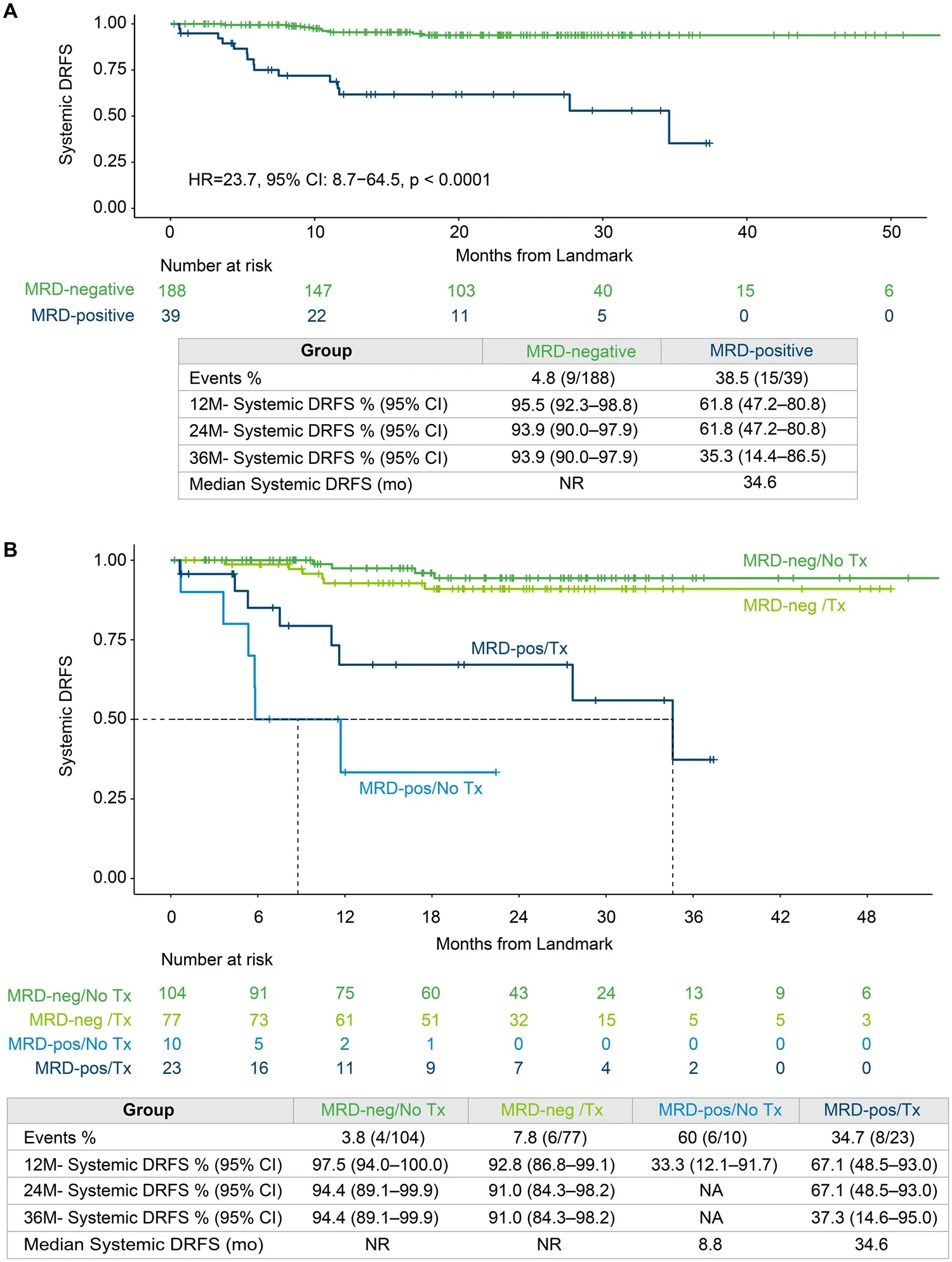
Prognostic and predictive value of MRD status at the landmark timepoint. (A,B) Kaplan–Meier estimates of systemic DRFS stratified by MRD status at the landmark timepoint alone (A) or in combination with adjuvant therapy status (B). HR, hazard ratio; MRD−, MRD-negative; MRD+, MRD-positive; systemic DRFS, systemic recurrence-free survival; Tx, adjuvant treatment.

### Association of longitudinal MRD status post-definitive treatment with systemic DRFS

In a pan-cancer analysis performed in a longitudinal setting (Figure 1B), ctDNA positivity rates demonstrated a significant stage-dependent upward trend, rising from 9.8% at Stage I to 15.5% at Stage II and 22.6% at Stage III (Cochran-Armitage trend test, *P* = .018). This increase was most pronounced when comparing Stage I to Stage III (Fisher’s exact, *P* = .030). Patients with MRD positivity at any time after definitive treatment were nearly 105 times more likely to experience a systemic recurrence compared to those with serial MRD negativity (HR: 103.4, 95% CI, 43.7-273.5, *P* < .0001; Figure 4A). To account for potential unmeasured heterogeneity across tumor types, a shared frailty term for cancer type was included in the model, with frailty variance estimated to be 0.22. Notably, 36-month systemic DRFS was 98.8% (97.2%-100%) for patients who remained serially MRD negative compared to only 14.7% (6.6-32.7%) for those with MRD positivity during post-definitive treatment surveillance. Tumor-specific analyses demonstrated similar findings in each of the subcohorts. MRD positivity post-definitive treatment was associated with significantly inferior systemic DRFS compared to those with serial MRD negativity in all cancer types evaluated: breast cancer (HR: 221.2, 95% CI, 131.0-373.4, *P* < .0001; Figure 4B), NSCLC (HR: 38.2, 95% CI, 10.5-138.6, *P* < .0001; Figure 4C), melanoma (HR: 55.9, 95% CI, 17.3-180.5, *P* < .0001; Figure 4D), RCC (HR: 104.7, 95% CI, 52.4-209.2, *P* < .0001; Figure 4E), and CRC (HR: 47.8, 95% CI, 30.1-76.0, *P* < .0001; Figure 4F). Signatera Genome provided a median lead time gain of 3 months in detecting relapses over the exome-based Signatera assay (data not shown).

**Figure 4.**
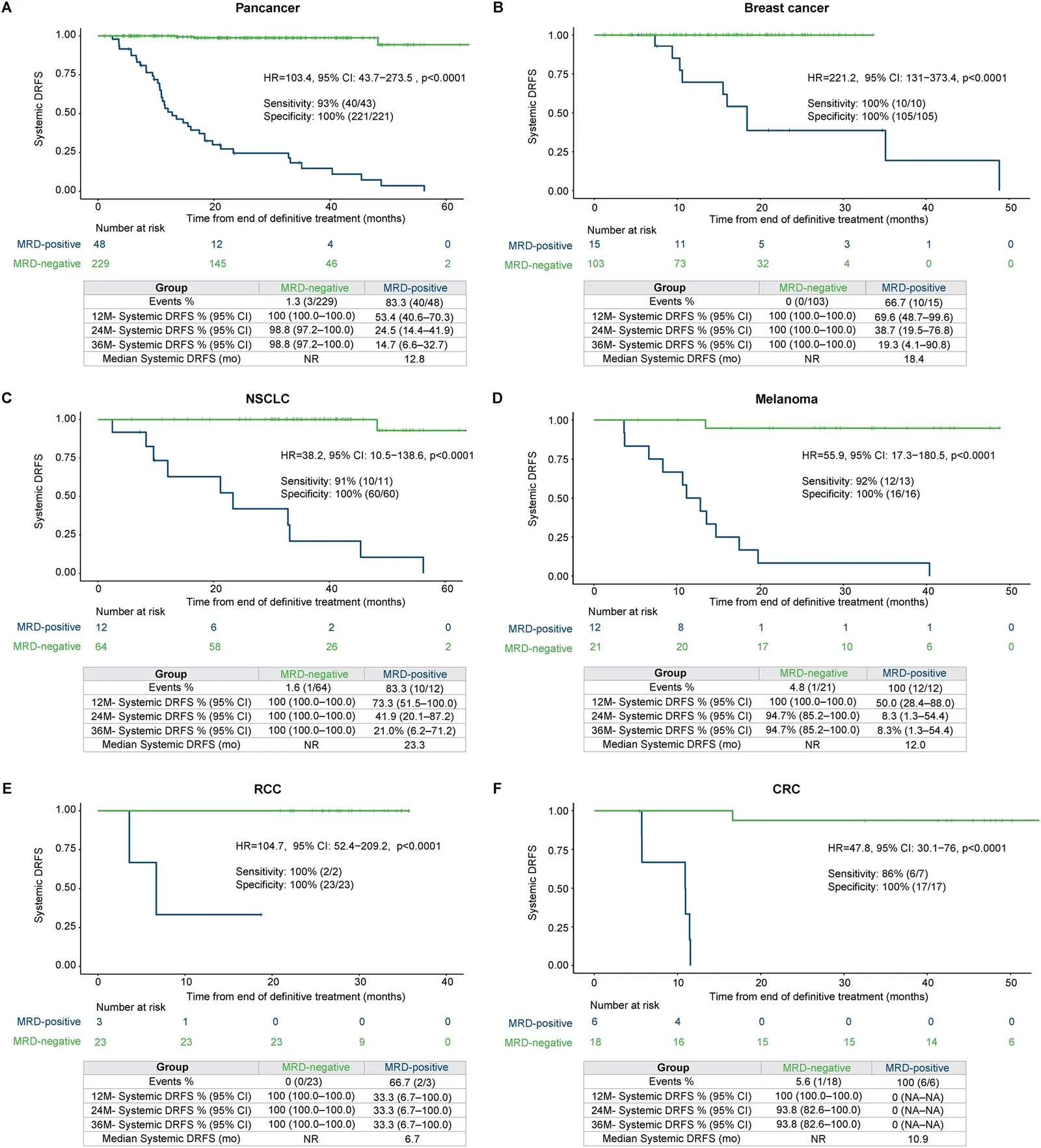
Prognostic value of longitudinal MRD status during post-definitive treatment surveillance. (A–F) Kaplan–Meier estimates of systemic DRFS stratified by longitudinal MRD status post-definitive treatment among the pan-cancer cohort (A), breast cancer (B), NSCLC (C), melanoma (D), RCC (E), and CRC (F) subcohorts. To account for potential unmeasured heterogeneity across tumor types, a shared frailty term for cancer type was included in the model. HR, hazard ratio; MRD−, MRD-negative; MRD+, MRD-positive; NSCLC, non-small-cell lung cancer; systemic DRFS, systemic recurrence-free survival; Tx, adjuvant treatment.

In the pan-cancer cohort, a clinical sensitivity of >90% and a specificity of 100% were observed in the longitudinal setting for detecting systemic recurrences, notably with a 100% sensitivity observed in the breast cancer and RCC subcohorts (Figure 4). Among patients who remained serially MRD negative (*N* = 229), locoregional or central nervous system (CNS)-only relapses and *de novo* primaries were noted in 7 (3%) patients (3 with NSCLC and 4 with melanoma).

## Discussion

WGS-based tumor-informed MRD assays are anticipated to offer an opportunity for enhanced sensitivity, offering a clear benefit for tumors characterized by low mutational burden or low ctDNA shedding and early disease-stage cancers. In contrast to WES, which targets roughly 1-2% of the genome, consisting of protein-coding exons, WGS evaluates the entire genome across both coding and non-coding regions. As a result, the WGS approach expands the pool of detectable patient-specific, somatic variants available for PCR amplicon design, thus allowing design of ctDNA assays with higher plex. This broader pool of candidate variants also enables more stringent thresholds for variant allele frequency and background error rate, ensuring that the tracked variants are of superior quality rather than just higher in quantity. Our findings underscore the robust analytical and clinical performance of the Signatera Genome assay for MRD detection across a spectrum of solid tumors. The assay demonstrated high clinical sensitivity across 5 cancer types, ranging from 86% to 100% for detecting systemic relapse in the longitudinal setting, and consistently delivered 100% specificity, reinforcing its reliability for clinical decision-making by minimizing false positives. Notably, Signatera Genome demonstrated the ability to identify the majority of relapses within a short period of time after surgery. In breast and lung cancer patients, ctDNA testing at the 3-month post-surgery timepoint identified those at risk of relapse with a sensitivity of 75% and 71%, respectively. Furthermore, a substantial number (∼50%) of samples, corresponding to 62% of the patients, were detected below 0.01% VAF (<100 ppm), indicating robust assay performance in the ultra-sensitive range. The expanded genomic footprint assessed by the WGS approach is expected to increase the assay sensitivity, which likely resulted in a longer observed lead time for ctDNA detection over radiographic imaging.

Previous studies using the WES-based Signatera assay within 3-month post-surgery demonstrated the prognostic value of MRD positivity, which was associated with significantly inferior patient outcomes in several of the individual tumor types included in our analysis: resectable CRC (disease-free survival [DFS]: HR: 11.99, *P* < .0001; OS: HR: 9.68, *P* < .0001),^4^ early breast cancer (DRFS: HR: 5.5, *P* = .043),^18^ clinical stage II-III NSCLC (RFS: HR: 4.18, *P* < .01; OS: HR: 14.4, *P* < .01),^19^ and melanoma (distant metastasis-free survival [DMFS]: HR: 10.77; *P* = .01).^5^ Our current findings build on this established evidence and demonstrate that ctDNA testing using the Signatera Genome assay also provides strong prognostic and predictive information at this early post-operative landmark across multiple solid tumor types. MRD positivity 3-month post-surgery was associated with a markedly increased risk of systemic recurrence (HR: 23.7; *P* < .0001), supporting the utility of early post-operative ctDNA testing for risk stratification. Importantly, MRD-negative patients had excellent outcomes, with nearly 94% systemic DRFS at 24 months, suggesting that the absence of detectable ctDNA defines a group with a favorable prognosis in whom treatment de-escalation may be possible.

Beyond its prognostic value, landmark ctDNA status also showed potential predictive relevance for adjuvant therapy benefit. Although limited by sample size, ctDNA-positive patients appeared to derive clinically meaningful benefit from adjuvant treatment, with substantial improvements in median and 24-month systemic DRFS, whereas ctDNA-negative patients showed no clear benefit from adjuvant therapy. These findings are consistent with the concept that ctDNA-based MRD assessment can risk-stratify patients and may support more personalized treatment decisions, intensifying therapy or switching to novel systemic therapies for those with molecular evidence of disease while sparing low-risk patients from overtreatment. Clinical trials utilizing WGS-based ctDNA assays, such as those evaluating MRD-guided de-escalation or escalation strategies, will be essential to formally integrate these findings into clinical guidelines.

The longitudinal analyses further underscore the strength of ctDNA, highlighting its value as a dynamic biomarker of recurrence risk. ctDNA positivity with the Signatera Genome assay at any time after definitive treatment was associated with an extraordinarily high risk of distant relapse across cancer types, even after accounting for inter-tumor heterogeneity (HR: 104.7, *P* < .0001). Serial ctDNA negativity identified a population with an exceptionally low risk of systemic recurrence, with nearly 99% systemic DRFS at 36 months, reinforcing the high negative predictive value of longitudinal ctDNA testing. The consistency of these findings across breast cancer, NSCLC, melanoma, RCC, and CRC highlights the broad applicability of this assay in diverse tumor contexts. Our findings supporting the utility of longitudinal ctDNA testing for post-treatment surveillance are in accordance with prior studies showing that WES-based Signatera detects recurrence across all of these tumor types, often months ahead of conventional imaging. In CRC, ctDNA positivity during surveillance was associated with significantly worse DFS (HR: 33.56, *P* < .0001) and OS (HR: 19.51, *P* < .0001),^4^ and in metastatic RCC, longitudinal ctDNA positivity off-treatment predicted significantly shorter PFS (HR: 18, *P* = .00026).^20^ Similarly, patients with ctDNA positivity during post-definitive treatment surveillance have been shown to experience significantly inferior RFS in melanoma (HR: 45.98, *P* < .0001)^21^ and in NSCLC (*P* < .0001).^22^ Whereas, in breast cancer,^7^^,^^13^ ctDNA positivity at any time post-surgery was associated with shorter RFS. Taken together, these data suggest that recurrence can be anticipated using longitudinal bespoke ctDNA monitoring, thus potentially enabling personalized post-treatment surveillance and earlier intervention in the recurrence setting.

Our study should be interpreted in the context of limitations typical of retrospective real-world analyses, including potential selection bias and unmeasured confounders; however, the inclusion of a diverse pan-cancer cohort reflects real-world clinical practice and strengthens the generalizability of the findings. Although sample sizes for certain tumor types, such as melanoma, CRC, and RCC, limited tumor-specific analyses, the consistent direction and magnitude of effect observed across cohorts support the robustness of the results. Variability in ctDNA testing intervals and adjuvant treatment decisions, driven by provider discretion, further underscores the real-world applicability of the data. However, incorporation of a shared frailty model helped account for inter-tumor heterogeneity in the pan-cancer analyses, providing a rigorous framework for evaluating ctDNA performance across biologically distinct cancer types. Finally, given the relatively short median follow-up (22.5 months), the analysis presented should be interpreted in the context of early relapses, since our dataset cannot fully capture the late relapse, particularly in HR+ breast cancer and in RCC.

## Conclusion

Signatera Genome represents a significant advancement in ctDNA-based MRD detection. The broad applicability across tumor types and high specificity of this ultra-sensitive assay support its integration into clinical practice and prospective trials. Further prospective studies are warranted to confirm clinical utility and facilitate wider adoption in early-stage disease management and surveillance paradigms, ultimately supporting incorporation into clinical guidelines for more personalized and effective cancer care.

## Data Availability

To protect the privacy and confidentiality of patients in this study, clinical data are not made publicly available but can be requested at any time from the corresponding author. All data shared will be de-identified and will be provided to researchers with access limited for scientific verification purposes and with strict prohibitions on secondary use.
